# Burkitt lymphomagenesis linked to MYC plus PI3K in germinal center B cells

**DOI:** 10.18632/oncotarget.726

**Published:** 2012-10-28

**Authors:** Sandrine Sander, Klaus Rajewsky

**Affiliations:** Immune Regulation and Cancer, Max-Delbrück-Center for Molecular Medicine, Berlin, Germany; Immune Regulation and Cancer, Max-Delbrück-Center for Molecular Medicine, Berlin, Germany

Burkitt lymphoma (BL) is a highly aggressive germinal center (GC) derived B cell lymphoma. Its successful treatment requires intense therapeutic protocols that include high doses of alkylating agents and central nervous system (CNS) prophylaxis [1]. Outcome is still poor in patients over the age of 40 years. Thus, less toxic and more targeted treatment strategies are needed to improve BL management, especially in developing countries, where BL occurs endemically and infrastructural shortages preclude appropriate treatment.

A better understanding of BL pathogenesis is a prerequisite for therapeutic advance. Activation of the *MYC* proto-oncogene at 8q24 via translocation to one of the immunoglobulin loci is typically detected in BL [2]. Despite driving proliferation and retarding differentiation, sole MYC overexpression is not sufficient for tumor development because of its associated apoptotic effects. Although many studies have focused on the oncogenic potential of MYC deregulation, the pro-survival signals co-operating with MYC in BL development remained elusive.

Based on comprehensive transcript analyses in human BL, revealing low NF-κB activity, [3,4] and the implication of PI3K in mature B cell survival [5], the PI3K pathway seemed a good candidate for providing the increased survival signal in BL development. We indeed found that transgenic mice in which combined MYC overexpression and constitutive activation of the PI3K pathway was targeted into GC B cells developed lymphomas strikingly resembling human BL [6]. The tumors in this model displayed not only a GC B cell like gene expression pattern, but also phenocopied their human counterparts with respect to location, histology, cell surface marker expression and other characteristics. Like most cases of BL diagnosed in the Western hemisphere, the mouse tumors predominantly presented as an abdominal mass stemming from the Peyer's patches. In addition, the murine lymphomas showed BL characteristic immunohistochemistry (BCL6^pos^, BCL2^neg^, Ki67>95%) and mRNA profiles (BL gene expression signature).

The most striking demonstration of the GC B cell origin of the BL-like tumors arising in the mutant mice was that of ongoing somatic hypermutation of the rearranged immunoglobulin variable gene segments in the tumor cells, as it is also observed in human BL. Former mouse models addressing the impact of MYC activation in B cell lymphomagenesis but nor targeting the event specifically into GC B cells, resulted in the generation of lymphomas originating from pre-GC B cells and resembling human BL only to limited extents [7-9].

The monoclonality of the tumors arising in mice upon MYC and PI3K pathway activation in GC B cells implies, that additional, tertiary mutations are needed for tumor development. Indeed, the tumors displayed unique genomic aberrations including trisomy of chromosome 6, which comprises genomic regions commonly gained in human BL, and somatic mutations of *Ccnd3* [6], a member of the D cyclin family essential for GC B cell proliferation. Notably, a recent analysis of human BL by transcriptome sequencing also reported *CCND3* mutations partly affecting the same codons as in the mouse tumors [10], a finding confirmed by Sander et al. (2012). Furthermore, the study by Schmitz and colleagues revealed mutations affecting the *TCF3*/*ID3* genes that result in consecutive B cell receptor upregulation and thus provide a potential mechanism for PI3K activation in human BL, which was indeed observed by both Schmitz and Sander et al. (2012).

In summary, we hypothesized cooperation between MYC and PI3K in BL development and functionally validated this hypothesis through conditional targeted mutagenesis in the mouse. This implicates the PI3K pathway in addition to MYC as a therapeutic target in BL. Additional therapeutic targets are suggested by tertiary mutations recurrently found in the mouse tumors and human BL. Ultimately, the in vivo model offers exciting opportunities to improve BL treatment by testing novel therapeutic strategies in the context of normal physiology, including a functional immune system. A particularly attractive perspective is the study of mechanisms controlling tumor metastasis, like the dissemination of BL into the CNS.
